# Maternal depression attenuates newborn vitamin D concentrations in winter-spring: a prospective population-based study

**DOI:** 10.1038/s41598-017-01778-1

**Published:** 2017-05-08

**Authors:** Qi-fan Zhou, Meng-xiao Zhang, Shi-lu Tong, Rui-xue Tao, Jia-hu Hao, Kun Huang, Fang-biao Tao, Peng Zhu

**Affiliations:** 1 Department of Maternal, Child & Adolescent Health, and Anhui Provincial Key Laboratory of Population Health & Aristogenics, Anhui Medical University, Hefei, China; 2 School of Public Health and social works, Queensland University of Technology, Kelvin Grove, Qld Australia; 3 Department of Gynecology and Obstetrics, Hefei First People’s Hospital, Hefei, China

## Abstract

We aimed to investigate whether the newborns of mothers with maternal depression (MD) had lower vitamin D levels than newborns of non-MD (NMD) mothers and identify the potential mechanism underlying this association. Maternal depressive symptoms in late pregnancy and concentrations of cord blood 25 hydroxyvitamin D (25(OH)D) were measured in 1491 mother-infant pairs. Data on maternal sociodemographic characteristics, health status, lifestyle and birth outcomes were prospectively collected. For infants born in winter-spring, the infants of MD mothers had significantly reduced concentrations of 25(OH) D (adjusted β = −3.51 nmol/L; 95% CI: −6.19, −0.84; *P* = 0.010) and lower birth weight (3267 ± 470 g vs 3348 ± 598 g, F = 4.64, *P* = 0.031), compared with the infants of NMD mothers. A significant, inverse linear relationship was noted between maternal depression scores and the concentration of 25(OH)D for infants born in winter-spring (adjusted β = −0.158; 95% CI: −0.259, −0.057). The significant, inverse linear relationship between maternal depression scores and fetomaternal ratios of 25(OH) D was also observed among the infants born in winter-spring (adjusted β = −0.005; 95% CI: −0.008, −0.003). MD appears to significantly attenuate the vitamin D concentrations and birth weight of infants born in winter-spring. A decreased fetomaternal ratio of 25(OH)D might be involved in this biological pathway.

## Introduction

Increasing evidence suggests that low vitamin D concentration in cord blood at birth may be an independent risk factor of adverse health outcomes in offspring, including elevated immune responses^1^, infection^2^, wheezing^3^ and mental disease^4^. Twenty-five hydroxyvitamin D [25(OH)D] is the major circulating form of vitamin D, which represents the cumulative effects of dietary intake of vitamin D and exposure to sunlight^5^. Maternal 25(OH)D is the only source of fetal vitamin D and correlates with neonatal concentrations at birth^6–8^.

Maternal depression (MD) is associated with adverse offspring development^9, 10^ and approximately 13% to 23% of pregnant women experience a depressive disorder^11, 12^. Recently, growing epidemiological evidence indicated that the pregnant women with depression have lower vitamin D levels compared with those without depression^13–15^. Additionally, the seasonality of both individual vitamin D levels and depressive symptoms have been observed in previous studies^16, 17^. Therefore, these convergent clues led to the new hypothesis that decreased levels of maternal and fetal vitamin D might be a potential biological pathway underlying the association between MD during pregnancy and adverse offspring development. To date, it is unclear whether the newborns of depressive mothers had lower vitamin D concentrations compared with newborns of non-depressive mothers. This concern is based on the strong relationship between maternal and fetal 25(OH)D levels^18^.

In this study, we examined the concentration of cord blood 25(OH) D in 1,491 neonates in Hefei, China and assessed the association between MD in late pregnancy and cord blood 25(OH) D concentrations at birth. The potential mechanism by which MD influences fetal vitamin D levels through disturbing maternal vitamin D concentrations or the fetomaternal ratio of vitamin D was also examined in this study.

## Results

Attrition analyses showed that the distributions of sociodemographic characteristics, health status, lifestyle and birth outcomes in non-participants did not differ from participants. In this study, there were 218 (14.6%) pregnant women above the clinically significant cutoff for depressive symptoms. The differences in demographics and clinical characteristics between mother-infant pairs with and without maternal depression were assessed using univariate analysis (Table 1). Depressive pregnant women had significantly reduced education attainment, family income, less gestational weight gain and gestational weeks at delivery. Alcohol consumption and husband smoking were significantly more often noted in depressive pregnant women compared with non-depressive pregnant women. The proportion of female infants among the offspring of depressive pregnant women was significantly reduced compared with non-depressive pregnant women. Additionally, newborns of MD were more likely to have reduced cord blood 25(OH)D concentrations and increased risk of vitamin D deficiency (less than 25 nmol/L).

**Table 1:** Demographics and clinical characteristics between mother-infant pairs with and without maternal depression.

Characteristics	Depressed (n = 218)	Non- depressed (n = 1273)	*P* value^a^
**Sociodemographic characteristics**			
Maternal age [Mean (SD)] (years)	27.22 (3.91)	27.73 (3.61)	0.059
Maternal education <9 years [*n* (%)]	63 (28.9)	247 (19.4)	0.001
Family monthly income <2000 yuan RMB [*n* (%)]	44 (20.2)	181 (14.2)	0.023
**Health status**			
Prepregnancy BMI [Mean (SD)] (kg/m^2^)	20.14 (2.22)	20.16 (2.45)	0.884
Gestational weight gain [Mean (SD)] (kg)	16.04 (4.73)	16.88 (4.87)	0.018
Multipara [*n* (%)]	33 (15.1)	166 (13.0)	0.400
Pregnancy complication^b^ [*n* (%)]	36 (16.5)	190 (14.9)	0.546
**Lifestyle**			
Maternal alcohol consumption^c^ [*n* (%)]	47 (21.6)	178 (14.0)	0.004
Paternal alcohol consumption^c^ [*n* (%)]	176 (80.7)	1023 (80.4)	0.898
Paternal smoking^d^ [*n* (%)]	72 (33.3)	264 (20.7)	<0.001
Vitamin D supplementation^e^ [*n* (%)]	100 (45.9)	612 (48.1)	0.547
**Birth outcomes**			
Female infant [*n* (%)]	100 (45.9)	691 (54.3)	0.022
Birth during winter-spring [n(%)]	110 (50.5)	589 (46.3)	0.252
Gestational weeks [Mean (SD)] (weeks)	38.6 (1.9)	39.0 (1.4)	0.008
Birth weight [Mean (SD)] (g)	3336 (549)	3393 (430)	0.149
Cord blood 25(OH)D [Mean (SD)] (nmol/L)	36.89 (20.93)	39.87 (20.23)	0.046
Cord blood 25(OH)D < 25 nmol/L [*n* (%)]	71 (32.6)	318 (25.0)	0.018

Abbreviation: 25(OH)D, 25-hydroxyvitamin D; BMI, body mass index.

^a^Chi-square test for categorical variables and Student’s t-test for continuous variables with univariate analysis.

^b^Complication of pregnancy included diabetes mellitus, hypertension, abnormal heart function, thyroid disease, intrahepatic cholestasis of pregnancy, and moderate and severe anemia.

^c^Maternal and paternal alcohol consumption was defined as any alcohol consumption up to 6 months before pregnancy.

^d^Paternal smoking was defined as more than 6 cigarettes daily up to 6 months before pregnancy.

^e^Maternal vitamin D supplementation was defined as the use of vitamin D supplement for greaterthan two months during pregnancy.

**Figure 1:**
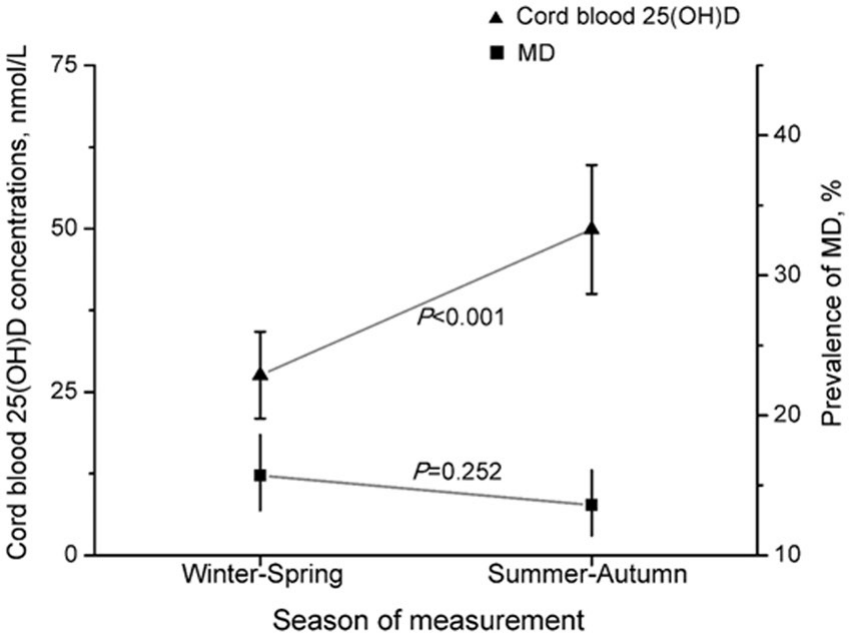
Maternal depression (MD) and newborn concentrations of 25(OH)D across the seasons. MD was defined as the scores of Center for Epidemiological Studies Depression Scale greater than a clinically significant cutoff score of 16. Bars represent the mean and 95% CI of the mean.

A significant difference in concentrations of cord blood 25(OH) D was noted between infants born in winter-spring and summer-autumn. Compared with infants born in summer-autumn, the 25(OH)D levels of infants born in winter-spring were significantly reduced (27.59 ± 13.30 nmol/L vs. 49.89 ± 19.78 nmol/L, *P* < 0.001). Correspondingly, the risk of vitamin D deficiency for infants born in winter-spring significantly increased (OR = 18.75; 95% CI: 13.20, 26.63; *P* < 0.001). The prevalence of MD measured in winter-spring (15.7%; 95% CI: 13.2, 18.6) was increased compared with that in summer-autumn (13.6%; 95% CI: 11.4, 16.1), but the difference was not statistically significant (Fig. 1).

**Figure 2:**
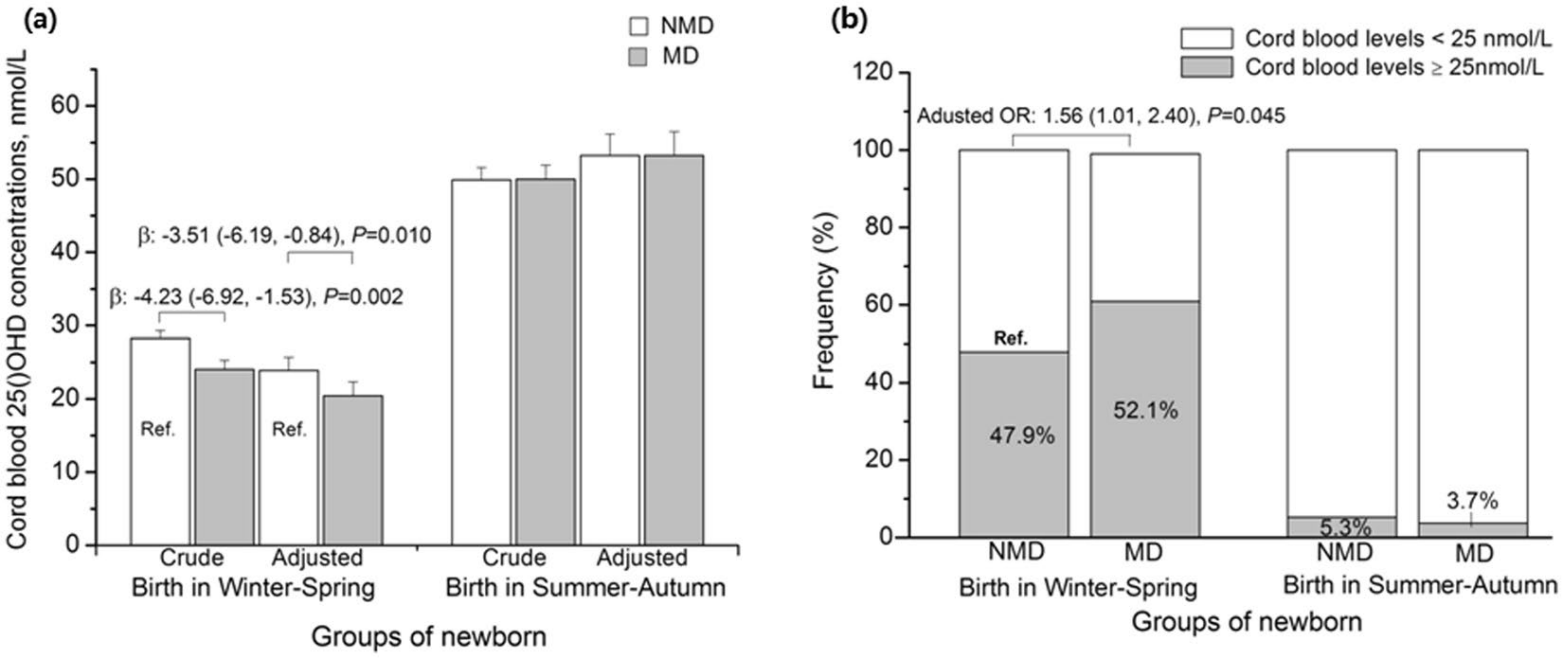
Associations between maternal depression (MD) and concentrations of cord blood 25(OH)D stratified by season. The differences of concentrations of cord blood 25(OH)D and the risks of 25(OH)D <25 nmol/L in the newborn of MD versus the newborn of non-MD were assessed using linear regression models and logistic regression models, respectively. Multiple regression models were adjusted for maternal sociodemographic characteristics, perinatal health status, lifestyle and birth outcomes. MD was defined as the scores of Center for Epidemiological Studies Depression Scale greater than a clinically significant cutoff score of 16. Bars represent the mean ± SE. OR, odd ratios; β, beta-coefficients.

Significant interactive effects of the scores on the Center for Epidemiological Studies Depression Scale (CES-D) and season of birth on the infant vitamin D levels were identified (F = 5.45, *P* = 0.020). We then stratified the data by season. For infants born in winter-spring, infants of mothers with MD had significantly reduced concentrations of cord blood 25(OH)D (adjusted β = −3.51 nmol/L; 95% CI: −6.19, −0.84; *P* = 0.010) and elevated risks of vitamin D deficiency (adjusted OR = 1.56; 95% CI: 1.01, 2.40; *P* = 0.045), compared with the infants of NMD mothers. However, the significant difference in concentrations of cord blood 25(OH) D (adjusted β = 0.32 nmol/L; 95% CI: −3.78, 4.42; *P* = 0.88) and elevated risk of vitamin D deficiency (adjusted OR = 0.75; 95% CI: 0.26, 2.19; *P* = 0.60) were not observed among infants born in summer-autumn (Fig. 2).

We conducted sensitivity analyses by adjusting for propensity scores in the multiple regression models. The difference in cord blood 25(OH)D concentrations (adjusted β = −3.91; 95% CI: −6.63, −1.18; *P* = 0.005) and the risk of vitamin D deficiency (adjusted OR = 1.60; 95% CI: 1.05, 2.44; *P* = 0.029) remained statistically significant for the infants of mothers with MD born in winter-spring, compared with the infants of NMD mothers. Additionally, when preterm births and infants with lower birth weight were excluded, this association remains statistically significant (adjusted β = −3.24; 95% CI: −6.15, −0.33; *P* = 0.029; adjusted OR = 1.15; 95% CI: 1.00, 1.33; *P* = 0.046).

**Figure 3:**
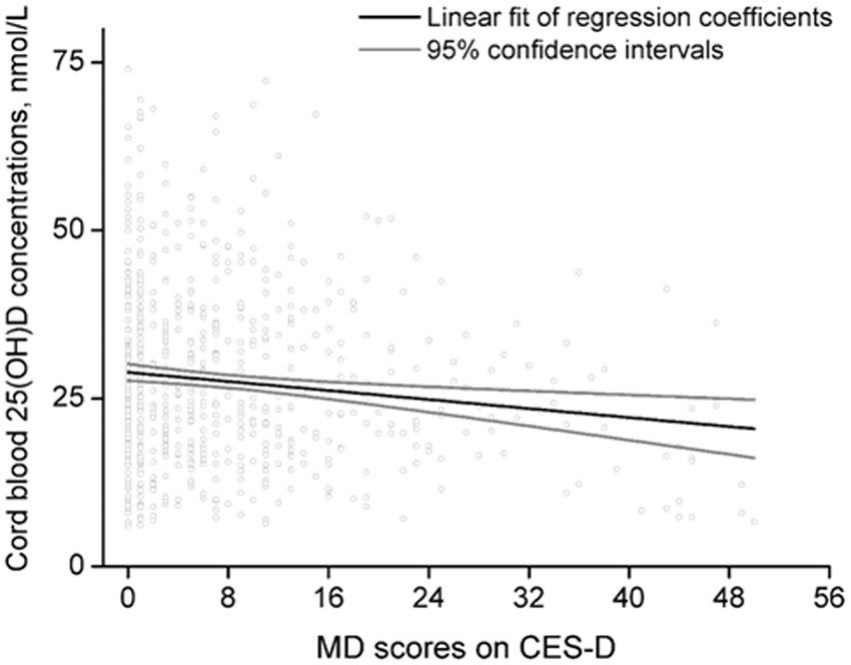
Linear relationship between maternal depression (MD) scores on CES-D and cord blood concentrations of 25(OH)D. The solid black line denotes the fit of the regression model; the solid grey line denotes the 95% CI. 25(OH)D, 25-hydroxyvitamin D; CES-D, Center for Epidemiological Studies Depression Scale.

Figure 3 presents a significant inverse relationship between maternal CES-D scores and the concentrations of cord blood 25(OH)D among infants born in winter-spring (adjusted β = −0.158; 95% CI: −0.259, −0.057; *P* = 0.002). This linear relationship is strengthened (adjusted β = −0.327; 95% CI: −0.529, −0.125; *P* = 0.002) among depressive pregnant women. Additionally, our results showed that the infants of MD mothers had significantly reduced birth weight compared with infants of NMD among infants born in winter-spring (3348 ± 598 g vs. 3267 ± 470 g, F = 4.64, *P* = 0.031), but not in summer-autumn (3320 ± 530 g vs. 3352 ± 445 g, F = 0.80, *P* = 0.37).

**Figure 4:**
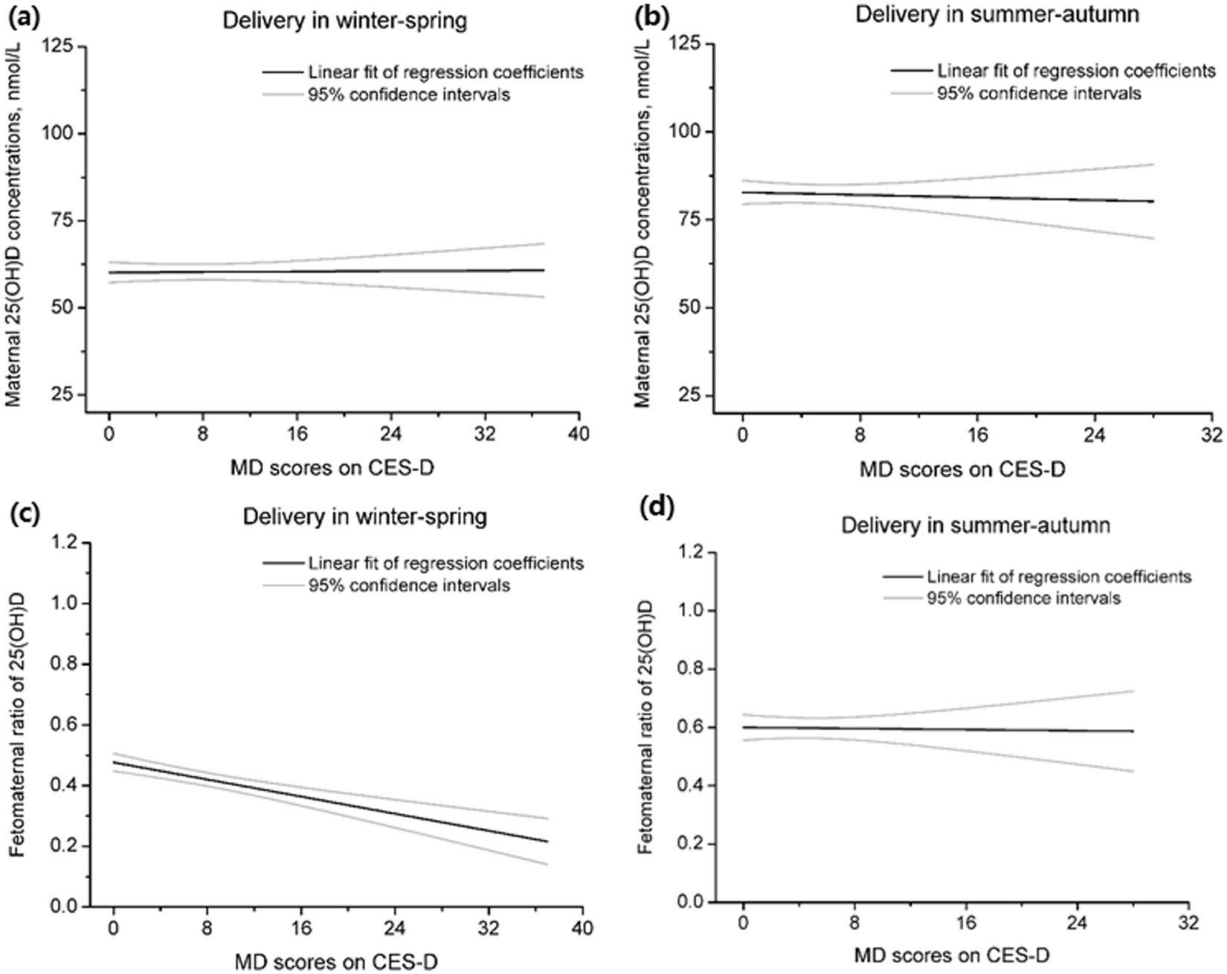
Linear associations of maternal depression (MD) score on CES-D with maternal 25(OH)D concentrations and the fetomaternal ratio of 25(OH)D concentrations. 25(OH)D, 25-hydroxyvitamin D; CES-D, Center for Epidemiological Studies Depression Scale.

Analysis of 466 mother-infant pairs with both maternal circulating blood before delivery and cord blood reveals a significant difference in maternal circulating 25(OH)D concentrations between winter-spring and summer-autumn (60.31 ± 17.14 nmol/L vs. 82.36 ± 20.56 nmol/L, *P* < 0.001). Unexpectedly, we did not observe a significant difference between depressive pregnant women and non-depressive pregnant women in maternal circulating 25(OH)D concentrations stratified by season. However, for infants born in winter-spring, the fetomaternal ratio of 25(OH)D among mother-infant pairs of MD mothers was significantly reduced compared with NMD mothers (0.35 ± 0.13 vs. 0.44 ± 0.19, *P* = 0.007). The significant inverse linear relationship between maternal CES-D scores and the fetomaternal ratio of 25(OH)D was also observed among mother-infant pairs born in winter-spring (adjusted β = −0.005; 95% CI: −0.008, −0.003; *P* < 0.001) but not those born in summer-autumn (Fig. 4).

## Discussion

Depending on the latitude, sunlight hours can differ substantially between seasons, and seasonal variation in vitamin D status was evident. Consistent with previous studies^19–21^, our results indicated that the concentrations of 25(OH)D in pregnant women and newborns were significantly reduced in winter-spring compared with summer-autumn. Furthermore, this study provided the first epidemiological evidence that for infants born in winter-spring, the infants of MD mothers had reduced vitamin D levels and birth weight compared with infants of NMD mothers.

To date, the guidelines concerning vitamin D screening in pregnant women are conflicting^16, 22–26^. The Committee Opinion of the American College of Obstetricians and Gynecologists^22^ and Australian Government antenatal care clinical practice guidelines^24^ recommend only screening women at increased risk of deficiency, including women with limited sun exposure^20^. As newborn vitamin D levels are largely dependent on maternal vitamin D status, the evidence provided in our study supports the recommendation to screen women who deliver their babies in winter-spring, especially when they suffer from prenatal depression.

The significant dose-response relationship between maternal depressive symptoms in late pregnancy and cord blood 25(OH)D levels at birth among mother-infant pairs born in winter-spring strengthens this association. However, to our knowledge, there is no research on the biological mechanism underlying this association. In this study, we further evaluated the potential biological pathway. Unexpectedly, the evidence of this study supports the hypothesis that the decreased fetomaternal ratio of 25(OH)D rather than decreased maternal 25(OH)D concentrations might be a potential biological pathway underlying the association. This finding raised the possibility that depressive pregnant women might need more vitamin D supplements, given their lower fetomaternal ratio of 25(OH)D.

It has been confirmed that 25(OH)D could readily traverse the hemochorial placentas^27^, such that cord blood 25(OH)D concentrations are equal to or less than maternal concentrations^28, 29^. It was assumed that the fetomaternal ratio of 25(OH)D might increase with pregnancy. As fetal vitamin D requirements increase (related to the increased need for fetal calcium), maternal vitamin D subsequently progressively decreased with pregnancy, especially if pregnancy occurs in winter^30^. Maternal depression might disturb vitamin D metabolism in the mother or fetus/placenta through affecting vitamin D-binding protein^18, 31^ and placenta-specific methylation of the CYP24A1 gene^32, 33^, which strongly regulates the production of 25(OH)D. Moreover, maternal depression might influences maternal 25(OH)D transportation across the placenta through altering placental activity and physiology, including the placental barrier, endocrine function and placental inflammation^34, 35^. However, high levels of maternal vitamin D in the summer-autumn^20^ may have masked the variation in the fetomaternal ratio of 25(OH)D, as decreased vitamin D induced by maternal depression is offset by the increased exposure to sunlight in summer-autumn. Further studies are warranted to explore the mechanism.

Our study did not identify a significant association between maternal depression and circulating 25(OH)D levels, which was observed in previous studies^13–15^. In our study, vitamin D concentrations were measured in late pregnancy rather than early pregnancy as performed in previous studies. Fetal vitamin D requirements increase during pregnancy; thus, maternal vitamin D levels tend to decreased during the third trimester^30^. The nature of maternal vitamin D levels during pregnancy might account for these inconsistent findings.

This study has four major strengths. First, it is the first observational epidemiological study on the association between maternal depression and newborn vitamin D status. Second, a cohort study design, relatively large sample size and prospective data collection procedure were used. Third, an inverse dose-response relationship between the maternal depressive symptoms and concentrations of cord blood 25(OH)D was evident, suggesting causality in this association. Finally, adjustment for a range of confounders, multiple sensitivity analyses and an exploration of a potential biological pathway also strengthen the findings in this study.

However, several limitations in this study should also be acknowledged. First, attrition bias was inevitable in this prospective study, particularly when maternal 25(OH)D concentrations were measured in only a small proportion of women. Second, the absence of data on exposure of sunlight and dietary intake was one of the major limitations, which may result in residual confounding in this study. Given that the effect size of sunlight and dietary intake on vitamin D levels are substantially greater compared with that of depression, caution should be warranted in understanding the role of MD on the maternal and fetal vitamin D levels. Third, this study was conducted in one city, and caution is necessary in generalizing the findings to other regions due to the strong influence of latitude.

In conclusion, infants born in winter-spring are at a high risk of vitamin D deficiency, and MD further attenuates vitamin D concentrations in these infants. A reduced fetomaternal ratio of 25(OH)D appears to be involved in the biological pathway. Clinically, this finding indicates that depressive pregnant women might require more vitamin D supplements due to their reduced fetomaternal ratio of 25(OH)D and infant birth weight. Pregnant women and their infants may benefit from screening for maternal depression and vitamin D supplementation.

## Methods

### Study populations

As part of the China-Anhui Birth Cohort (C-ABC) study^36^, 2,552 pregnant women who received prenatal check-ups in Hefei Maternal and Child Health Hospital from January to September 2008 were recruited in Hefei (32°N latitude). Pregnant women at gestational ages from 30 to 34 weeks, participated in this study and completed a structured questionnaire, including sociodemographic characteristics, lifestyle and the CES-D. Maternal non-fasting blood samples were collected within one week before delivery, and cord blood was collected at birth by study nurses when available. Data on pregnancy history, pregnancy complications, and delivery outcomes were obtained through the interviews or medical records. The C-ABC study was conducted according to the Declaration of Helsinki guidelines. The Ethics Committee of Anhui Medical University granted ethical approval. All experiments in this study were performed in accordance with the relevant guidelines and regulations and written informed consent was obtained from each participant.

In this study, to exclude potential confounding factors, stillbirth, birth defects, women with delivery before 32 weeks of gestation (early preterm), pregnancy with assisted reproductive technology, or multiple gestations were excluded from the study. Finally, we obtained 1,491 available mother-infant pairs with cord blood and 466 mother-infant pairs with both maternal circulating blood before delivery and cord blood.

### Maternal depressive symptoms

Maternal depressive symptoms in late pregnancy were assessed using the Chinese version of the 20-item Center for Epidemiological Studies Depression Scale (CES-D) with well-established reliability^37^. CES-D was designed to assess the levels of depressive symptomatology within the previous week with a clinically significant cutoff score of 16 or greater. Each item is scored on a 4-point scale of frequency of occurrence of the symptom, ranging from rarely or none of the time (0) to most or all the time (3). Internal consistency (Cronbach’s alpha) of the scales was 0.86.

### 25-hydoxyvitamin D in maternal circulation and cord blood

Plasma samples were centrifuged and promptly refrigerated at −4 °C within 12 h and transferred to −80 °C freezers. Concentrations of 25(OH)D were measured using commercial radioimmunoassay kits (DiaSorin Stillwater, MN, USA). Intra-assay and inter-assay coefficients of variation were 8.8% and 11.1%, respectively. Cord blood concentrations of 25(OH)D were analyzed as both continuous and dichotomous variables with a cutoff of 25 nmol/L for vitamin D deficiency as recommended by the Canadian Paediatric Society^38^.

### Potential confounders

Information on major confounders, including maternal sociodemographic characteristics, perinatal health status, lifestyle, birth outcomes and seasons, was prospectively collected from medical records or interviews. Maternal sociodemographic characteristics included age, education (≤ 9 and >9 years of completed schooling), and household income (less than 2,000 and greater than 2,000 RMB Yuan/month). Perinatal health status included prepregnancy body mass index (BMI), gestational weight gain, parity (nulliparous or multiparous), and pregnancy complications. Pre-pregnancy BMI was calculated based on the height routinely measured at the clinic visit and on the prepregnancy weight obtained at interview. The amount of gestational weight gain (GWG) was determined by subtracting the prepregnancy weight from the measured weight recorded at the last prenatal visit before delivery. Pregnancy complications included diabetes mellitus, hypertension, abnormal heart function, glandular thyroid disease, intrahepatic cholestasis of pregnancy, and moderate and severe anemia. Perinatal lifestyle included maternal alcohol consumption, paternal smoking and alcohol consumption up to 6 months before pregnancy, and maternal vitamin D supplementation during pregnancy. Birth outcomes included gestational age and birth weight. The gestational age (in completed weeks) based on the difference between the date of the last menstrual period and the date of delivery and was categorized as <37 (preterm birth) or ≥37 gestational weeks. Low birthweight was defined as an infant weighing less than 2,500 g. The season was designated as: winter (December, January, February), spring (March, April, May), summer (June, July, August), or autumn (September, October, November).

### Statistical analysis

Chi-square test or Student’s t-test analyses were adopted to test the differences in the demographics and clinical characteristics of depressive pregnant women and non-depressive pregnant women. The differences in the prevalence of maternal depression and concentrations of cord blood 25(OH)D between winter-spring and summer-autumn were analyzed using Student’s t-test or the Chi-square test.

Stratified by season, we examined the differences in concentrations of cord blood 25(OH)D and infants birth weight between depressive pregnant women and non-depressive pregnant women using a multiple linear model. The risk of vitamin D deficiency for newborns of MD mothers was assessed using a multiple logistic regression model after adjustment for confounders. We though that the analyses with both a dichotomous variable for vitamin D deficiency and a continuous variable of 25(OH)D could strengthen the results and suggest more clinical significance. A range of potential confounders, which were potentially associated with maternal depressive symptoms or 25(OH)D levels in this study or previous studies^20, 39^, were adjusted in multiple regression models, including demographic characteristics, such as maternal age, education and family income; perinatal health status, such as prepregnancy BMI, GWG, parity, and pregnancy complication; perinatal lifestyle, such as maternal alcohol consumption, vitamin D supplementation, paternal alcohol consumption and smoking; and birth outcomes, such as infant gender, gestational weeks, birth weight and birth season.

Sensitivity analyses were performed after adjustment for the propensity score as a continuous variable or a categorical variable with four levels. The propensity score method is a technique for controlling confounding in a multiple model. Unlike conventional statistical approaches that depend on a model of the outcome under study, the propensity score method relies on a model of the exposure^40^. Furthermore, we additionally restricted our analyses to healthy newborns without preterm birth (born between 32 and 37 gestational weeks) and low birth weight, to avoid confounding by adverse birth outcomes. The dose-response relationships between MD and the cord blood 25(OH)D levels were assessed using multiple linear regression models after adjustment for confounders. Additionally, we compared the difference in the birth weight between infants of MD and NMD mothers.

We further tested the differences in maternal 25(OH)D concentrations and fetomaternal ratios of 25(OH)D between depressive pregnant women and non-depressive pregnant women using multiple linear regression models. We performed all analyses using SPSS (version 21.0, IBM Corp: Armonk, NY, USA). All analyses were two-tailed, and *P* < 0.05 was considered statistically significant.
